# Atypical processing of voice sounds in infants at risk for autism spectrum disorder

**DOI:** 10.1016/j.cortex.2015.06.015

**Published:** 2015-10

**Authors:** Anna Blasi, Sarah Lloyd-Fox, Vaheshta Sethna, Michael J. Brammer, Evelyne Mercure, Lynne Murray, Steven C.R. Williams, Andrew Simmons, Declan G.M. Murphy, Mark H. Johnson

**Affiliations:** a Birkbeck, University of London, Centre for Brain and Cognitive Development, UK; b King's College London, Institute of Psychiatry, Psychology & Neuroscience, Sackler Institute of Translational Neurodevelopment, Department of Forensic and Neurodevelopmental Science, UK; c University College London, Institute of Cognitive Neuroscience, UK; d University of Reading, School of Psychology and Clinical Language Sciences, UK; e Stellenbosch University, South Africa; f NIHR Biomedical Research Centre for Mental Health at South London and Maudsley NHS Foundation Trust and King's College London Institute of Psychiatry, UK

**Keywords:** Autism, Brain imaging, Infant development, Social interaction, Voice processing, ASD, autism spectrum disorder, fMRI, functional magnetic resonance imaging, HR, high risk, LR, low risk, BA, Broadman area, HRF, haemodynamic response function

## Abstract

Adults diagnosed with autism spectrum disorder (ASD) show a reduced sensitivity (degree of selective response) to social stimuli such as human voices. In order to determine whether this reduced sensitivity is a consequence of years of poor social interaction and communication or is present prior to significant experience, we used functional MRI to examine cortical sensitivity to auditory stimuli in infants at high familial risk for later emerging ASD (HR group, N = 15), and compared this to infants with no family history of ASD (LR group, N = 18). The infants (aged between 4 and 7 months) were presented with voice and environmental sounds while asleep in the scanner and their behaviour was also examined in the context of observed parent–infant interaction. Whereas LR infants showed early specialisation for human voice processing in right temporal and medial frontal regions, the HR infants did not. Similarly, LR infants showed stronger sensitivity than HR infants to sad vocalisations in the right fusiform gyrus and left hippocampus. Also, in the HR group only, there was an association between each infant's degree of engagement during social interaction and the degree of voice sensitivity in key cortical regions. These results suggest that at least some infants at high-risk for ASD have atypical neural responses to human voice with and without emotional valence. Further exploration of the relationship between behaviour during social interaction and voice processing may help better understand the mechanisms that lead to different outcomes in at risk populations.

## 1 Introduction

One of the basic foundations for social communication is the human voice, which is arguably the most important acoustic stimulus in an individuals' social environment as it carries important cues such as speaker identity and emotional state. Further, research with adults has revealed that cortical regions along the superior temporal sulcus (STS) show stronger activation when participants listen to human vocalisations (speech, laughter, crying, coughing, etc.) as compared to non-vocal environmental sounds and acoustically matched stimuli (Belin, Zatorre, Lafaille, Ahad, & Pike, 2000). Activation of these temporal voice-selective areas can also be modulated by emotional information carried on the voice (Grandjean et al., 2005), as can activation in other areas such as inferior prefrontal cortex (Fecteau, 2005), premotor cortical regions (Warren et al., 2006) and the amygdala (Fecteau, Belin, Joanette, & Armony, 2007), insula and orbitofrontal cortex (Chikazoe, Lee, Kriegeskorte, & Anderson, 2014). Hence there is compelling evidence that specific regions of the human brain respond to voice and emotional voice sounds.

One important question, however, is how the network of specialized regions tuned to social information emerges in the developing human brain. Addressing this question is crucial not only to better understand typical development, but also to increase our understanding of disorders that involve impaired development of social cognition, such as autism spectrum disorders (ASD). Functional neuroimaging studies by our group and others have revealed that from early infancy the typically developing brain is tuned to perceive and process information carried by the voice (Dehaene-Lambertz, Dehaene, & Hertz-Pannier, 2002; Lloyd-Fox, Blasi, Mercure, Elwell, & Johnson, 2012), and can be modulated by emotions (Grossmann, 2010). In a previous study we addressed the issue of the emergence of specialized brain regions for processing the human voice (Blasi et al., 2011) by investigating the brain responses to adult non-speech vocalisations (emotionally neutral, emotionally positive, and emotionally negative) and non vocal sounds in a group of typically developing infants (aged between 3 and 7 months) asleep in the MRI scanner. Our results showed an early functional specialisation for processing the human voice, with significant differential activation to vocal sounds (compared to non-vocal sounds) in the anterior portion of the temporal cortex [similarly to the findings in adults (Belin et al., 2000)], and also in the medial frontal gyri. In addition, we compared the brain responses to vocal sounds with positive (laughter) and negative (crying) valence to neutral vocal sounds and we found that sad vocalisations modulated the activity of brain regions involved in processing affective stimuli such as the orbitofrontal cortex (Kringelbach, 2005) and insula (Morris, Scott, & Dolan, 1999), whereas there was no differential response between happy and neutral vocalisations. These results point toward an emergence of specialisation of brain regions for processing stimuli that enable communication and learning of social behaviour. The data collected in our previous study has contributed to the LR group in the current study with the exception of three participants who had to be excluded from the current analysis (see the Methods section).

As ASD are characterised by deficits in social communication and behaviour, it is of paramount interest to investigate further when these deficits emerge in the process of development. Based on the possibility that one cause of the deficit in communication in ASD is an underlying atypical perception of sensory stimuli (C.R.G. Jones et al., 2009), we hypothesised that infants at-risk of later ASD may not show the early specialisation for processing the human voice. Auditory processing in the context of ASD has been extensively investigated with neurophysiological techniques such as event-related potentials (ERPs) which, thanks to their high temporal resolution, can reveal stimulus-specific neural responsiveness (see the reviews by O'Connor, 2012 and Kujala, Lepistö, & Näätänen, 2013). These studies have shown that both children and adults with ASD present an enhanced proficiency in processing low-level auditory stimuli (such as tones), however this advantage is lost when the complexity of the stimuli increases (O'Connor, 2012), affecting their ability to learn and understand language (Lepistö et al., 2008). These effects are reflected in the anatomical distribution of the responses to speech stimuli across age ranges in the context of ASD, with reduced activation in the left temporal and frontal regions (regions typically associated with language processing). Further, it has also been reported that these deficits in the left hemisphere may be compensated for by enhanced dominance of the right hemisphere (O'Connor, 2012). Right hemisphere dominance in ASD may be associated with enhanced proficiency in processing spectral characteristics of auditory stimuli, whereas left hemisphere deficiencies may be associated with diminished performance in processing temporal aspects of auditory stimuli with direct effect on speech perception (Haesen, Boets, & Wagemans, 2011). In the present work we focus on information about the human voice without the complexities of speech and language.

One particular area of interest for the analysis of voice stimuli is the extraction of information regarding emotions. Although many studies of brain function have addressed processing emotional facial expressions in the context of ASD (e.g., see Stewart, McAdam, Ota, Peppe, & Cleland, 2013), relatively few have examined the processing of socially relevant auditory information. Those which are available (e.g., Gervais et al., 2004) have reported that when presented with voice and non voice sounds, neurotypical adults showed stronger activation to voice compared to non voice stimuli whereas those with ASD did not. Further, no significant differences were reported between the groups in the responses to non voice sounds. In addition, and related to the evidence for atypical voice processing, adults and children diagnosed with ASD show difficulty in recognising the emotions of others when the information is conveyed by acoustic-prosodic stimuli (Golan, Baron-Cohen, Hill, & Rutherford, 2006; Stewart et al., 2013). In summary, there is strong evidence showing that individuals with ASD have atypical processing of social and emotional stimuli. However, the developmental time course of these atypicalities is unclear.

In order to better understand how, when and where developmental trajectories that result in ASD deviate from the typical, several research groups have studied infant siblings of older children diagnosed with ASD (E.J.H. Jones, Gliga, Bedford, Charman, & Johnson, 2014; Gliga, Jones, Bedford, Charman, & Johnson, 2014), as around 20% of these infants will go on to a later diagnosis themselves (Ozonoff et al., 2011). With the first overt behavioural symptoms appearing only toward the end of the first year of life, affected infants will typically not be routinely diagnosed before their third birthday (E.J.H. Jones et al., 2014). However, results from infant sibling studies suggest that the underlying differences in brain function that later on give rise to the behavioural symptoms may already be evident during the first year of life (Elsabbagh et al., 2012; Lloyd-Fox et al., 2013). Despite this, to our knowledge, no studies have directly investigated brain response to human vocal sounds in infants at high risk of ASD. Yet such studies could provide crucial evidence on the onset of the disorder as, according to the Interactive Specialisation perspective on typical development (M.H. Johnson, 2000), cortical areas that become tuned to social stimuli develop through a process of reinforcement by differential patterns of experience. Disruption of this process may arise due to an atypical developmental trajectory compounded by later atypical interactions with the environment, which may ultimately lead to the well-established profile of ASD symptoms by the age of diagnosis. Moreover, for the developing infant, an important canalisation of environmental experience is through the interaction with their primary caregiver. Therefore, an increasing number of studies have suggested that the nature of this interpersonal interaction may be a sensitive early indicator of later problems (for a review, see E.J.H. Jones et al., 2014), and could provide an important context for a more complete understanding of the disorder (Elsabbagh & Johnson, 2010). However, relatively little is known on how atypical interaction patterns influence children's neurobiological development (Taylor, Eisenberger, Saxbe, Lehman, & Lieberman, 2006), and to our knowledge, there have been no studies that have investigated the moderating role of infant and parent behaviours on the association between risk status and brain activation.

Given the current lack of evidence, this study utilized fMRI to examine differences in brain response to human vocalisations in sleeping 4–7-month old infants. Infants with no family (first degree relative) history of ASD (LR group) were compared with infants with at least one full sibling with a community clinical diagnosis of ASD (HR group). Three specific questions were asked: first, are there differences in voice processing between HR and LR infants?; second, is there a group difference in the infant's sensitivity to affect (i.e., sad emotions) in vocal sounds?; and third, is variation in parent–child interaction associated with differences in infant brain responsivity?

## 2 Material and methods

### 2.1 Participants

fMRI data were acquired from a group of 33 infants at the Centre for Neuroimaging Sciences of the Institute of Psychiatry, Kings College London. 15 of the infants had at least one full sibling with a community clinical diagnosis of ASD (HR group, 147 ± 25 days of age, 10 male). These participants were within the average range of functioning (mean 96.8, standard deviation 9.86) as measured by the Early Learning Composite (ELC) standard scores of the Mullen Scales of Early Learning (Mullen, 1995). HR infants were recruited via the British ASD Study of Infant Siblings (BASIS), a UK collaborative network facilitating research with infants at risk for ASD that also provided ethical approval and informed consent, as well as background data on participating families. The remaining 18 participants had no family (first degree relative) history of ASD and had all been included in our previous work [(Blasi et al., 2011); LR group, 154 ± 26 days of age, 7 male]. 3 infants from the original LR group of 21 had to be excluded from the current study as one received an ASD diagnosis after the first publication, and two had incomplete fMRI data sets, with 4 and 6 trials missing (out of a total of 32) at the end of the run. Exclusion was necessary on this second ground as the transformation step required for the group comparisons needs complete experimental data sets for the calculations. As a result, the data of the remaining 18 LR participants were re-analysed after smoothing, at the end of the pre-processing sequence (see detailed description of the data analysis in the Supplemental Experimental Procedures section of the Supplementary Material). Infants in the low- and high-risk groups were of similar age (independent samples *t*-test, *p* = .464, *t* = .753); and Mullen ELC standard scores (only available from 6 of the LR infants: mean 105, standard deviation 7.34) were also similar (*p* = .076, *t* = 1.869).

As part of a multi-centre project, this research was also approved by the Institute of Psychiatry and South London and Maudsley Research Ethics Committee.

### 2.2 Experimental design

#### 2.2.1 Risk status: exposure

High-risk status was defined by having an older sibling with a community clinical diagnosis of ASD confirmed by two expert clinicians based on the Development and Wellbeing Assessment (DAWBA, Goodman, Ford, Richards, Gatward, & Meltzer, 2000) and the parent-report Social Communication Questionnaire (SCQ, Rutter, Bailey, & Lord, 2003).

#### 2.2.2 fMRI data acquisition: outcome

Details of the experimental design are described in our previous publication (Blasi et al., 2011). In brief, while naturally asleep in the scanner (without sedation) the infants were presented with three categories of adult non-speech vocalisations by different male and female speakers: emotionally neutral (yawning, sneezing or coughing), emotionally positive (laughter), and emotionally negative (crying) sounds. The infants were also presented with non-vocal environmental sounds with which they were likely to be familiar (toys and running water, hereafter referred as non voice). The stimuli were organized in a block design, in which 21 sec of auditory stimuli were alternated with 9 sec of rest. A complete fMRI session comprised 32 blocks (8 in each stimulus category) lasting a total of 16 min.

The MRI data were acquired on a clinical GE 1.5 T Twinspeed MRI scanner (General Electric, Milwaukee, WI, USA) equipped with an 8-channel head radiofrequency (RF) coil array. Details of the scanning sequences can be found in Blasi et al. (2011) and in the Supplemental Information.

#### 2.2.3 Measures of maternal and infant behaviour in the context of mother–infant interaction: moderator

Mothers participated in a laboratory based face-to-face play session for 5 min, within two weeks of the MRI session. Mother-infant interactions were video-recorded using a standard assessment protocol (Murray, Fiori-Cowley, Hooper, & Cooper, 1996). Mothers were asked to play with and talk to their infant (seated facing the mother) as they would normally, without the use of toys. Using the Global Rating Scales (Murray et al., 1996), four maternal (i.e., sensitivity, intrusiveness, remoteness and depressive affect) and 3 infant behavioural dimensions (i.e., attentiveness, active-engagement, and fretfulness) were coded (Supplementary Table 1 of the supplemental information), by two trained coders, blind to infant risk status. Inter-rater intraclass correlations (ICC) on a randomly selected 20% of the interactions ranged from .75 to .90, indicating acceptable inter-rater reliability. Measures of maternal and infant behaviours in the context of mother–infant interaction were available for the 18 LR infants with fMRI data and for 13 of the 15 HR infants with fMRI data.

### 2.3 Data analysis

#### 2.3.1 fMRI data analysis

We analysed the MRI data with XBAM (www.brainmap.co.uk/xbam.htm) using a data-driven approach based on the standard general linear model adjusted to incorporate the potential differences between adult and infant HRF (Richter & Richter, 2003). Instead of the standard adult HRF, for each participant, we used the mean HRF estimated from all the other participants (regardless of group), thus producing the best estimate of the HRF unbiased by the participant being analysed (see details in Blasi et al., 2011), assuming that there are no significant differences in the HRF between the groups (Feczko et al., 2012). We then analysed the data for each individual infant using standard GLM analysis and the estimated unbiased HRF.

The selection of the condition contrasts used for group comparisons was based on the results reported in our previous publication on a group of typically developing infants (Blasi et al., 2011). This narrowed down the contrasts to the following: (1) neutral voice versus non voice contrasts (neutral voice > non-voice; non voice > neutral voice); and (2) sad voice versus neutral voice contrasts (sad voice > neutral voice; neutral voice > sad voice).

*Between group* comparisons of the condition contrasts of interest revealed the clusters where group differences in voice processing were significant. However, this analysis did not provide information regarding the origin of the group differences, i.e., whether it was one group showing a stronger preference for one type of sound, or whether one group had a stronger preference for one type of sound whereas the other group showed preference for the other type of sound. In order to find out the origin of the group differences we extracted the betas (averaged across all voxels in a cluster of interest defined in the whole brain analysis) for each contrast (voice > non voice, non voice > voice, etc…) per participant. Then, the betas of the condition contrasts averaged across participants within each group were used as estimates of the group effect size in that cluster and, therefore, allowed us to identify the origin of the group difference.

#### 2.3.2 Moderation of behaviour during mother–infant interaction on the associations between risk-status and infant processing of vocal sounds

Moderation analyses were conducted on the contrasts that are related to social communication: neutral voice stronger than non voice (voice selectivity) and sad voice stronger than neutral voice (modulation of sad valence on the response to vocal sounds). Further, the regions of interest were selected from the list defined by the clusters with significant group differences (as the results of the fMRI data analysis indicated, see Table 1). We hypothesized that moderation of behaviour would occur in the regions typically reported in association with processing voice (Belin et al., 2000; Blasi et al., 2011), emotions (Peelen, Atkinson, & Vuilleumier, 2010) and forming part of the social brain network (Adolphs, 2003). Therefore, the following regions were selected from the voice selectivity contrast: left middle temporal gyri (clusters 2 and 16), left temporal lobe (cluster 17), left superior and medial frontal gyri (clusters 27 and 37) and right medial frontal gyri (clusters 32 and 39); and for the sad voice modulation contrast: right fusiform gyrus (cluster 4), and the left hippocampus (cluster 10). For each cluster, multiple linear regression models were constructed which included each participant's averaged beta value (as outcome) and an interaction term between *group* status and each *behavioural dimension*. FDR correction for multiple comparisons was applied to the results (Benjamini & Yekutieli, 2001).

## 3 Results

### 3.1 Voice processing in HR and LR infants (neutral voice *vs* non voice contrast)

#### 3.1.1 Within group activations

**Fig. 1:**
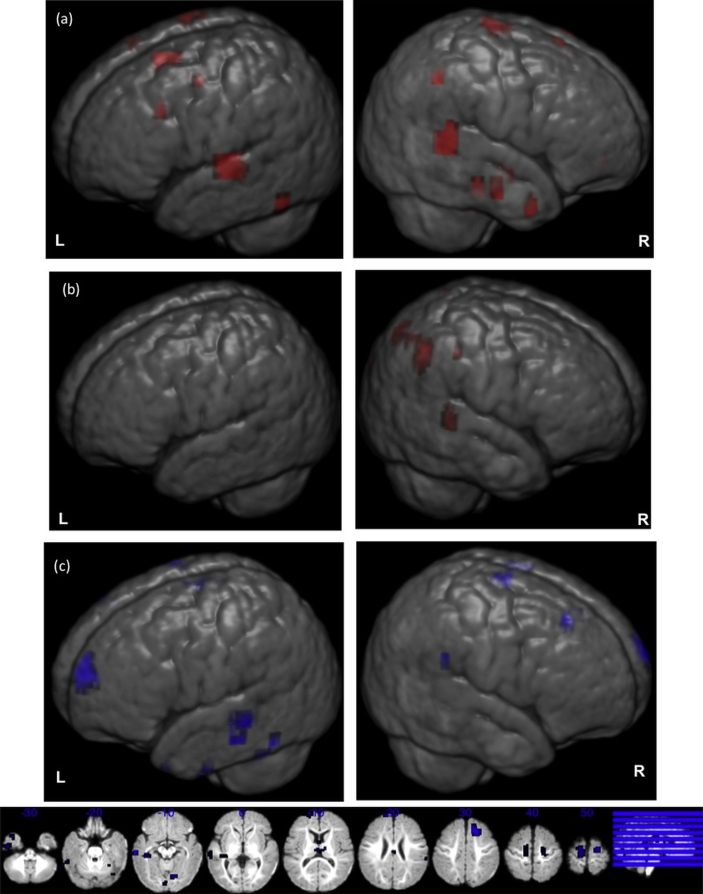
Neutral voice greater than non voice contrast. Representation on an age-appropriate infant template (Sanchez, Richards, & Almli, 2012) of the neutral voice greater than non voice condition contrast. (a) Low risk group, (b) high risk group, (c, d) group differences in the condition contrast; (L) left hemisphere, and (R) right hemisphere. See also Supplementary Tables 2 and 3

Infants in the low-risk group showed significantly stronger responses to the neutral voice condition as compared to the non voice condition (voice selectivity), bilaterally in the superior and middle temporal gyrus, in the superior and middle frontal gyrus, and also in the right cingulate gyrus. By contrast, infants in the high-risk group preferentially activated to neutral voice over the non voice condition, only in the right inferior parietal lobule and (similarly to the low-risk group) in a region of the right cingulate gyrus (Fig. 1a and Supplementary Table 2).

In both groups, brain functional response for non-vocal sounds over vocal sounds was significant in the left temporal gyrus. Additionally, infants in the high-risk group showed significant preference for non-vocal over neutral vocal sounds in the left cerebellum and the right pre-central gyrus (Fig. 1b and Supplementary Table 3).

#### 3.1.2 Between group differences

There were significant differences in voice selectivity in the left middle temporal gyrus and, bilaterally, in the superior/medial frontal gyri. Additionally, there were group differences in the left thalamus and caudate and right cerebellum (Fig. 1c and Table 1). Specifically, in the clusters with significant group differences, these were mainly due to different preference for voice over non voice conditions: whereas the low-risk infants showed stronger preference for voice (positive sign of the averaged beta values for the contrast voice *vs* non voice), the high-risk infants showed a tendency to respond more to non voice compared to voice (negative sign of the averaged beta values for the contrast voice *vs* non voice).

There were no significant group differences in brain response to the non voice over neutral voice conditions.

### 3.2 Sensitivity to sad affect in voice in HR and LR infants (sad voice *vs* neutral voice contrasts)

#### 3.2.1 Differences within group

In the analyses of sensitivity to sad affect in voice (sad voice > neutral voice) the low-risk infants showed significantly stronger responses to sad compared to neutral voice in the left superior frontal gyrus and the right inferior frontal gyrus; whereas the high-risk infants showed activation to sad affect in a small cluster within the right cingulate gyrus (8 voxels) (Supplementary Table 4). With reference to the neutral voice greater than sad voice contrast, low-risk infants showed a stronger activation to neutral vocal sounds in the left middle frontal gyrus, right superior temporal gyrus and the right uncus, whereas high-risk infants, showed greater activation to neutral vocal sounds bilaterally in the fusiform gyrus (with more clusters in the right hemisphere), the right lingual gyrus, middle frontal gyrus and left precentral gyrus (Supplementary Table 5).

#### 3.2.2 Differences between groups

**Fig. 2:**
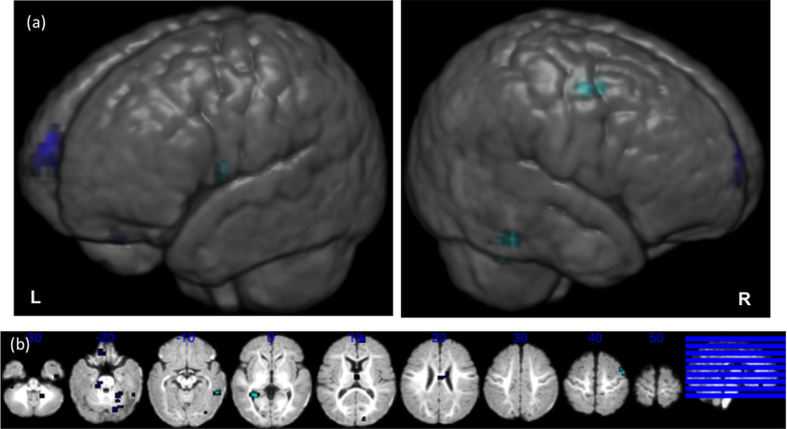
Neutral voice versus sad voice group differences. Representation on an age-appropriate infant template (Sanchez et al., 2012) of the between group differences in neutral voice versus sad voice contrast. Significant clusters with responses to sad voices stronger than to neutral voices are represented in cyan; significant clusters where response to neutral voice > sad voice are represented in blue. (a) Three-dimensional rendering of the group differences. (b) Results on slices of the same template. See also Table 2.

In the analyses of sensitivity to sad affect in voice (sad voice > neutral voice) the low-risk infants showed stronger activation than high-risk infants to sad vocal sounds in the right fusiform gyrus and left hippocampus (Table 1 and Fig. 2). High-risk infants did not activate significantly more than the low-risk in any brain region. With reference to the neutral voice greater than sad voice contrast, group differences (mostly low-risk infants showing stronger activation than high-risk infants) were found bilaterally in the caudate, and the right superior frontal gyrus (Table 1 and Fig. 2).

### 3.3 Moderation by maternal and infant interactive behaviours on the associations between risk-status and infants processing of vocal sounds

**Fig. 3:**
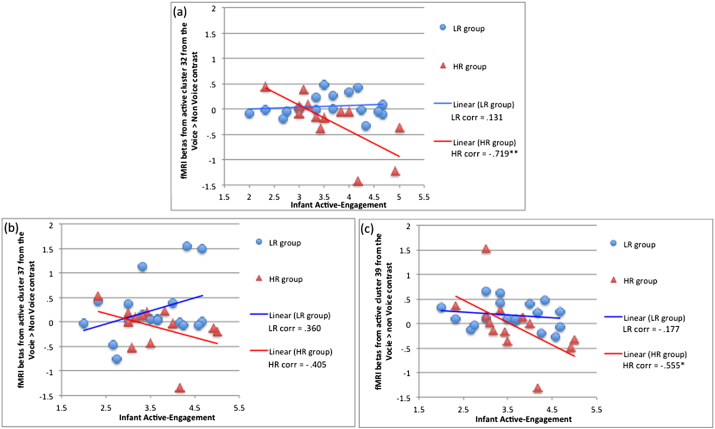
Association between behaviour in the context of mother–infant interaction and fMRI activation. Representation of the interaction between the infant behavioural measure Active-Engagement and group status on the voice sensitivity contrast in clusters (a) 32 (left medial frontal gyrus, BA 9); (b) 37 (left medial frontal gyrus, BA 6); and (c) 39 (right medial frontal gyrus, BA 6). Pearson correlation coefficients between Infant Active-Engagement and fMRI activation were calculated within group at each cluster; * and ** indicate significant Pearson correlation (2-tailed, at *p* < .05 level and *p* < .01 level, respectively).

For the contrast neutral voice > non voice, there were significant interactions between maternal and infant behaviours with risk status to predict infant processing of vocal sounds in a number of brain regions. Maternal intrusiveness × risk status predicted activation in the left middle temporal gyrus (cluster 16, BA 21), whereas infant behaviours (attentiveness, fretfulness and active-engagement) interacted with risk status to predict activation in the medial frontal gyrus (clusters 32, 37 and 39, as summarised in Table 2). However, the only effect that survived FDR correction for multiple comparisons was infant active-engagement × risk status in cluster 32 (voice selectivity contrast), in the right medial frontal gyrus (BA 9). Similar trends in the interaction between infant active-engagement and risk status were observed in the other two clusters in the medial frontal gyrus (as shown in Fig. 3). In these three clusters, infants in the HR group show negative correlation between active-engagement and voice selectivity: in cluster 32, Pearson correlation = −.719, *p* = .006 (2-tailed); in cluster 37, Pearson correlation = −.405, *p* = .170 (2-tailed); and in cluster 39, Pearson correlation = −.555, *p* = .049 (2-tailed). By contrast, infants in the LR group do not show any correlation between active-engagement and voice selectivity: in cluster 32, Pearson correlation = .131, *p* = .603 (2-tailed); in cluster 37, Pearson correlation = .360, *p* = .142 (2-tailed); and in cluster 39, Pearson correlation = −.177, *p* = .484 (2-tailed). Therefore, infants in the HR group with higher interaction scores on the active-engagement dimension (characterised by high levels of engagement, attentiveness and activity) tend to show weaker activation to voice sounds compared to non voice sounds; whereas, LR infants show a clear preference for vocal sounds, irrespective of infant behaviour (Fig. 3). There were no significant differences in measures of active-engagement between the two groups (LR, mean = 3.64, SD = .76; HR, mean = 3.60, SD = .77; comparison of means, *t* = .171, *p* = .865), and variance of this measure was also similar in the two groups [F(17,12) = 2.394, *p* = .935].

In contrast, neither maternal nor infant behaviours moderated the group differences found for the sad voice versus neutral voice fMRI contrasts.

## 4 Discussion

### 4.1 Voice-processing in HR and LR infants

In this fMRI study, infants in the high-risk group show a striking atypicality in human voice selectivity. Whereas low-risk infants show a clear pattern of stronger activation to voice sounds compared to non-voice sounds, in the middle and superior temporal regions, as well as the medial frontal gyrus, infants in the high-risk group show significantly *less* voice selectivity in these regions. Importantly, however, the two groups did not differ in non voice sound selectivity.

The results in the low-risk group are consistent with previously published research with adults (Belin et al., 2000) and with infants of similar age, (Grossmann, Oberecker, Koch, & Friederici, 2010; Lloyd-Fox et al., 2012). Adding to these previous studies, we have established that between 4 and 7 months there is already voice specialisation along the STS (similarly to that described in adults), but also in other brain regions such as the inferior frontal and fusiform cortex. As infants develop, the network of regions specialised in voice processing becomes more efficient, it narrows and consolidates in the temporal cortex (M.H. Johnson, 2011; Leppänen & Nelson, 2008), possibly freeing the frontal areas to be involved in higher level processing and expanding to the posterior part of the STS. Moreover, the diminished voice selectivity we found in 4–7-month old infants in the high-risk (compared to the low-risk group) is very similar to the responses found in adults: for instance, Gervais et al. (2004) report that adults with an ASD diagnosis show deficits in voice selectivity in similar cortical areas. Therefore our results are in line with those that suggest that an atypical cortical processing of socially relevant auditory information is already present in at risk infants from 4 to 7 months (Lloyd-Fox et al., 2013). In the present study, the use of fMRI has allowed us exploration of the specialisation for voice processing in the whole brain, while previous studies were restricted to responses in the surface cortical regions covered by the fNIRS sensor. In both fNIRS and fMRI studies, there is a clear reduction in voice selectivity in the group of high-risk infants, but a similar pattern of non voice selectivity in both groups of infants. This compelling consistency across sessions and imaging modalities further supports the hypothesis of an atypical processing of auditory stimuli in infants at risk for later emerging ASD.

The group differences in voice selectivity we observed were mainly located in the left hemisphere in a region often associated with language processing (Hickok & Poeppel, 2007). Previous fMRI research has also found reduced activation of the frontal-temporal regions to speech-related stimulation in ASD, sometimes coupled with increased activation in the right frontal regions to facilitate processing of auditory stimulation (O'Connor, 2012). These findings have been reported from very early in development [at 2–3 years of age (Redcay & Courchesne, 2008)], and they have been shown to increase with age, becoming more pronounced in 3–4 year olds with autism (Eyler, Pierce, & Courchesne, 2012). Although we did not find the compensatory hyper-responsivity in the right frontal region in our HR group (O'Connor, 2012; Redcay & Courchesne, 2008), possibly due to the young age of our participants and/or to the non-speech nature of our stimuli, our current findings raise the possibility that atypical voice processing from early infancy may be one of the contributing factors influencing disruption of the typical developmental trajectory of language acquisition (Lepistö et al., 2008). Our results are also in line with the Interactive Specialisation framework discussed earlier (M.H. Johnson, 2011), and a resulting lack of emerging specialisation of social brain regions in ASD. The Interactive Specialisation perspective on brain development views the process of emergence of the adult pattern of cortical specialisation as a progressive tuning of responses in certain cortical areas to social stimuli. According to this view, biases in attention and processing in early infancy are reinforced by differential patterns of subsequent experience, with the end result being the patterns of cortical specialisation associated with the social functions observed in adults. Therefore, the disruption of the mechanisms that bias infants to attend socially relevant mechanisms may, in turn, disrupt the typical trajectory that leads to the adult social brain network (Dawson et al., 2005; M.H. Johnson, 2011; Lloyd-Fox et al., 2012; Schultz, 2005).

In addition to the temporal regions, the low-risk infants also showed increased voice selectivity bilaterally in the medial frontal gyrus as compared to the high-risk infants. It has been suggested (Mundy, 2003) that impairment of this region and in the anterior cingulate may constitute a substrate for socio-cognitive deficits in ASD, as they both play a role in joint attention and other higher complex behaviours involving interaction with others. Regions of the frontal cortex have been reported to have an atypical overgrowth (Carper & Courchesne, 2005) and, possibly, an abnormal connectivity (Courchesne & Pierce, 2005) in children diagnosed with ASD. Hence, it is possible that atypical function of these regions may result in difficulties in the integration of information that gives relevance to vocal sounds that are then processed in the voice temporal regions. If correct, this disruption may also contribute to the diminished voice selectivity observed in our work (Haesen et al., 2011; O'Connor, 2012).

### 4.2 Sensitivity to sad affect in voice in HR and LR infants

In the analyses of possible differences between groups in the modulation of emotion on the brain responses to voice sounds, we found [similar to our previous publication (Blasi et al., 2011)] that this modulation was limited in both groups. This may be explained, in part, by our participants being asleep–as cortical activation in the response to auditory stimuli is reduced during sleep (Czisch, 2002). Therefore, it is possible that the differential brain activation between two vocal conditions in our sleeping participants may have been too subtle to detect. Nevertheless, significant group differences in sad voice modulation were found in the right fusiform gyrus and in the left hippocampus, with the low-risk participants showing stronger sad voice over neutral voice responses than the high-risk infants. Deficits in the amygdala-fusiform network, which support the development of face perception and social cognitive skills, may be instrumental in emerging ASD, as the development of social perceptual skills during childhood provides important scaffolding for social skill development (Schultz, 2005).

Atypical brain processing of socially relevant information may be linked with differences in behaviour during a highly social task such as mother–infant interaction. Therefore, we also investigated potential moderation effects of the interaction between group status and mother or infant behaviours in the context of mother–infant interactions observed within two weeks of the MRI scan.

### 4.3 Moderation by maternal and infant interactive behaviours

We found that the association between risk status and infant processing of voice in the right medial frontal gyrus was moderated by infant behaviour, characterised by active-engagement during observed mother–infant interactions. This finding suggests that group differences in brain responsivity can be accounted for, in part, by differences in social experience, which in turn are possibly created by the infants themselves. Moreover, we found a marginally significant group effect on one of the measures of maternal behaviour during mother–infant interaction: mothers of HR infants tended to display sad affect whilst interacting with their infant (M = 3.91, SD = .54), compared to mothers of LR infants (M = 4.27, SD = .46), although the difference was at trend level only (*t* = −2.0, *p* = .058). Therefore, it is possible that the infant's behaviour is driving the interaction in a way that the mother tends to modify her contribution to it in turn. Further, individual differences in infant behaviour require consideration, as these can potentially reflect different developmental pathways to outcome (Elsabbagh & Johnson, 2010). For instance, differences in temperament (characterised by lower activity levels and disengagement of visual attention) in some infants who later go on to develop ASD, have been reported in prospective studies (Zwaigenbaum et al., 2005). However, how differences in infant behaviour interact with risk to influence brain responsivity remains unknown, and the directionality in the mutual influences between mother and infant cannot be fully resolved from the current study.

In this study infant active-engagement was not independently associated with risk status or with brain activation. Yet, it did interact with risk status to predict brain response to non-vocal sounds, in HR infants. That is, HR infants who are more engaged in their early interactions show a tendency to respond more strongly in the region of the medial frontal gyrus to non vocal sounds compared to human voices. It is possible that this counterintuitive result is a manifestation of a protective trait of the infants who grow up and do not develop ASD, showing that their stronger responses to non voice sounds may counteract the deficit in processing the human voices we have found associated with the HR status. Future work is required, however, to determine if modulating the early behaviours of infant HR siblings alters their developmental brain trajectories.

A weakness of our study is that the high-risk infants have not yet been assessed for ASD at three years of age. While only a minority of our infants at-risk will go on to a later diagnosis of ASD, the unaffected siblings of children with ASD often share common patterns of atypical activation (“trait activity”) in cortical regions engaged in social processing, including the right inferior temporal gyrus, as reported in Kaiser et al. (2010). Thus, it is possible that our current results reflect “trait” activity in our high-risk infant group that will result in a later diagnosis of ASD only when combined with other genetic, neural, or environmental factors.

## Figures and Tables

**Table 1:** Group differences in brain activation. Clusters with significant group differences in voice-sensitivity (neutral voice > non voice), and sensitivity to sad affect (sad voice > neutral voice, and neutral voice > sad voice). In the last column, ‘+’ represents within group neutral voice > non voice; ‘−‘ represents within group neutral voice < non voice. BA = Broadman area, Num voxels = number of voxels in each cluster.

Cluster ID		BA	Tal(x)	Tal(y)	Tal(z)	Num voxels	Effect	LR versus HR
**Neutral voice** > **Non voice**								
2	L middle temporal gyrus	38	−36.11	11.11	−40.15	4	.012847	LR > HR (+/−)
16	L middle temporal gyrus	21	−57.78	−11.11	−12.65	14	.003296	LR > HR (+/−)
17	L temporal lobe (sub-gyral)	20	−36.11	−11.11	−18.15	7	.007975	LR > HR (+/−)
19	L thalamus		−10.83	−18.52	−1.65	4	.00815	LR > HR (+/+)
26	L caudate		−7.22	3.7	9.35	10	.004348	LR > HR (−/−)
27	L superior frontal gyrus	10	−21.67	66.67	9.35	12	.001486	LR > HR (−/−)
32	R medial frontal gyrus	9	10.83	40.74	31.35	24	.002501	LR > HR (+/−)
37	L medial frontal gyrus	6	−14.44	7.41	53.35	27	.006788	LR > HR (+/−)
39	R medial frontal gyrus	6	10.83	3.7	53.35	20	.006617	LR > HR (+/−)
**Sad voice** > **Neutral voice**								
4	R fusiform gyrus	20	54.17	−18.52	−23.65	11	.002187	LR > HR (+/+)
6	L hippocampus		−28.89	−25.93	−7.15	7	.000788	LR > HR (+/+)
**Neutral voice** > **Sad voice**								
11	R caudate		10.83	25.93	−1.65	5	.001707	LR < HR (−/+)
16	R superior frontal gyrus	10	7.22	66.67	−1.65	11	.00232	LR > HR (+/+)
17	L caudate		−3.61	11.11	9.35	5	.002646	LR > HR (+/−)

**Table 2:** Associations between group × maternal or infant behaviour predicting brain activation. Moderation analysis of mother–infant interaction behaviour measures and group status (LR or HR) on fMRI activations for the neutral voice > non voice contrast. Correlation coefficient (ΔR^2^) of the model, b and beta values (b, Beta), *t*-statistic (*t*) and *p*-values (*p*) of the moderation analysis for each component of the model are reported.* indicates *p* < .05; ** indicates *p* < .005.

	ΔR^2^	b(SE)	Beta	t	*p*
**Cluster 16 (L middle temporal gyrus):**					
	.284				
Group		−1.264 (.513)	−2.010	−2.463	.020
Maternal sensitivity		−.089 (.092)	−.201	−.963	.344
Maternal sensitivity × group		.308 (.154)	1.581	2.004	.055
	.349				
Group		−1.025 (.342)	−1.621	−3.0	.006
Maternal intrusiveness		−.24 (.062)	−.076	−.382	.705
Maternal intrusiveness × group		.240 (.100)	1.300	2.407	.023*
**Cluster 32 (R medial frontal gyrus):**					
	.558				
Group		1.044 (.601)	1.298	1.737	.094
Infant fretfulness		.007 (.087)	.016	.083	.934
Infant fretfulness × group		−.352 (.153)	−1.703	−2.293	.030*
	.435				
Group		.686 (.393)	.853	1.744	.093
Infant attentiveness		−.040 (.083)	−.096	−.482	.633
Infant attentiveness × group		−.301 (.122)	−1.287	−2.471	.020*
	.705				
Group		1.658 (.527)	2.062	3.143	.004
Infant inertness		.034 (.090)	.067	.383	.705
Infant active-engagement × group		−.539 (.144)	−2.498	−3.741	.001**
**Cluster 37 (L medial frontal gyrus):**					
	.322				
Group		1.133 (.606)	1.003	1.871	.072
Infant attentiveness		.378 (.129)	.643	2.938	.007*
Infant attentiveness × group		−.487 (.188)	−1.482	−2.595	.015*
	.216				
Group		1.489 (.925)	1.318	1.610	.119
Infant inertness		.266 (.157)	.369	1.693	.102
Infant active-engagement × group		−.511 (.253)	−1.685	−2.022	.053
	.463				
Group		1.489 (.902)	1.318	1.652	.110
Infant fretfulness		.148 (.131)	.237	1.134	.267
Infant fretfulness × group		−.477 (.230)	−1.647	−2.075	.048*
**Cluster 39 (R medial frontal gyrus):**					
	.287				
Group		1.240 (.716)	1.352	1.731	.095
Infant inertness		−.058 (.122)	−.099	−.476	.638
Infant active-engagement × group		−.399 (.196)	−1.620	−2.307	.052

## References

[bib1] Adolphs R. (2003). Cognitive neuroscience: cognitive neuroscience of human social behaviour. Nature Reviews Neuroscience.

[bib2] Belin P., Zatorre R.J., Lafaille P., Ahad P., Pike B. (2000). Voice-selective areas in human auditory cortex. Nature.

[bib3] Benjamini Y., Yekutieli D. (2001). The control of the false discovery rate in multiple testing under dependency. Annals of Statistics.

[bib4] Blasi A., Mercure E., Lloyd-Fox S., Thomson A., Brammer M., Sauter D. (2011). Early specialization for voice and emotion processing in the infant brain. Current Biology.

[bib5] Carper R.A., Courchesne E. (2005). Localized enlargement of the frontal cortex in early autism. Biological Psychiatry.

[bib6] Chikazoe J., Lee D.H., Kriegeskorte N., Anderson A.K. (2014). Population coding of affect across stimuli, modalities and individuals. Nature Neuroscience.

[bib7] Courchesne E., Pierce K. (2005). Why the frontal cortex in autism might be talking only to itself: local over-connectivity but long-distance disconnection. Current Opinion in Neurobiology.

[bib8] Czisch M. (2002). Altered processing of acoustic stimuli during sleep: reduced auditory activation and visual deactivation detected by a combined fMRI/EEG study. NeuroImage.

[bib9] Dawson G., Webb S.J., Wijsman E., Schellenberg G., Estes A., Munson J. (2005). Neurocognitive and electrophysiological evidence of altered face processing in parents of children with autism: implications for a model of abnormal development of social brain circuitry in autism. Development and Psychopathology.

[bib10] Dehaene-Lambertz G., Dehaene S., Hertz-Pannier L. (2002). Functional neuroimaging of speech perception in infants. Science.

[bib11] Elsabbagh M., Johnson M.H. (2010). Getting answers from babies about autism. Trends in Cognitive Sciences.

[bib12] Elsabbagh M., Mercure E., Hudry K., Chandler S., Pasco G., Charman T. (2012). Infant neural sensitivity to dynamic eye gaze is associated with later emerging autism. Current Biology.

[bib13] Eyler L.T., Pierce K., Courchesne E. (2012). A failure of left temporal cortex to specialize for language is an early emerging and fundamental property of autism. Brain.

[bib14] Fecteau S. (2005). Sensitivity to voice in human prefrontal cortex. Journal of Neurophysiology.

[bib15] Fecteau S., Belin P., Joanette Y., Armony J.L. (2007). Amygdala responses to nonlinguistic emotional vocalizations. NeuroImage.

[bib16] Feczko E., Miezin F.M., Constantino J.N., Schlaggar B.L., Petersen S.E., Pruett J.R. (2012). The hemodynamic response in children with simplex autism. Developmental Cognitive Neuroscience.

[bib17] Gervais H., Belin P., Boddaert N., Leboyer M., Coez A., Sfaello I. (2004). Abnormal cortical voice processing in autism. Nature Neuroscience.

[bib18] Gliga T., Jones E.J.H., Bedford R., Charman T., Johnson M.H. (2014). From early markers to neuro-developmental mechanisms of autism. Developmental Review.

[bib19] Golan O., Baron-Cohen S., Hill J.J., Rutherford M.D. (2006). The “Reading the Mind in the Voice” test-revised: a study of complex emotion recognition in adults with and without autism spectrum conditions. Journal of Autism and Developmental Disorders.

[bib20] Goodman R., Ford T., Richards H., Gatward R., Meltzer H. (2000). The development and well-being assessment: description and initial validation of an integrated assessment of child and adolescent psychopathology. Journal of Child Psychology and Psychiatry.

[bib21] Grandjean D., Sander D., Pourtois G., Schwartz S., Seghier M.L., Scherer K.R. (2005). The voices of wrath: brain responses to angry prosody in meaningless speech. Nature Neuroscience.

[bib22] Grossmann T. (2010). The development of emotion perception in face and voice during infancy. Restorative Neurology and Neuroscience.

[bib23] Grossmann T., Oberecker R., Koch S.P., Friederici A.D. (2010). The developmental origins of voice processing in the human brain. Neuron.

[bib24] Haesen B., Boets B., Wagemans J. (2011). A review of behavioural and electrophysiological studies on auditory processing and speech perception in autism spectrum disorders. Research in Autism Spectrum Disorders.

[bib25] Hickok G., Poeppel D. (2007). The cortical organization of speech processing. Nature Reviews Neuroscience.

[bib26] Johnson M.H. (2000). Interactive specialisation perspective on typical development. Child Development.

[bib27] Johnson M.H. (2011). Interactive specialization: a domain-general framework for human functional brain development?. Developmental Cognitive Neuroscience.

[bib28] Jones E.J.H., Gliga T., Bedford R., Charman T., Johnson M.H. (2014). Developmental pathways to autism: a review of prospective studies of infants at risk. Neuroscience & Biobehavioral Reviews.

[bib29] Jones C.R.G., Happé F., Baird G., Simonoff E., Marsden A.J.S., Tregay J. (2009). Auditory discrimination and auditory sensory behaviours in autism spectrum disorders. Neuropsychologia.

[bib30] Kaiser M.D., Hudac C.M., Shultz S., Lee S.M., Cheung C., Berken A.M. (2010). Neural signatures of autism. Proceedings of the National Academy of Sciences.

[bib31] Kringelbach M.L. (2005). The human orbitofrontal cortex: linking reward to hedonic experience. Nature Reviews Neuroscience.

[bib32] Kujala T., Lepistö T., Näätänen R. (2013). The neural basis of aberrant speech and audition in autism spectrum disorders. Neuroscience & Biobehavioral Reviews.

[bib33] Lepistö T., Kajander M., Vanhala R., Alku P., Huotilainen M., Näätänen R. (2008). The perception of invariant speech features in children with autism. Biological Psychology.

[bib34] Leppänen J.M., Nelson C.A. (2008). Tuning the developing brain to social signals of emotions. Nature Reviews Neuroscience.

[bib35] Lloyd-Fox S., Blasi A., Elwell C.E., Charman T., Murphy D., Johnson M.H. (2013). Reduced neural sensitivity to social stimuli in infants at risk for autism. Proceedings of the Royal Society B: Biological Sciences.

[bib36] Lloyd-Fox S., Blasi A., Mercure E., Elwell C.E., Johnson M.H. (2012). The emergence of cerebral specialization for the human voice over the first months of life. Social Neuroscience.

[bib37] Morris J.S., Scott S.K., Dolan R.J. (1999). Saying it with feeling: neural responses to emotional vocalizations. Neurophysiologia.

[bib38] Mullen E.M. (1995). Mullen scales of early learning.

[bib39] Mundy P. (2003). Annotation: the neural basis of social impairments in autism: the role of the dorsal medial-frontal cortex and anterior cingulate system. Journal of Child Psychology and Psychiatry.

[bib40] Murray L., Fiori-Cowley A., Hooper R., Cooper P. (1996). The impact of postnatal depression and associated adversity on early mother-infant interactions and later infant outcome. Child Development.

[bib41] Ozonoff S., Young G.S., Carter A., Messinger D., Yirmiya N., Zwaigenbaum L. (2011). Recurrence risk for autism spectrum disorders: a Baby Siblings Research Consortium Study. Pediatrics.

[bib42] O'Connor K. (2012). Auditory processing in autism spectrum disorder: a review. Neuroscience & Biobehavioral Reviews.

[bib43] Peelen M.V., Atkinson A.P., Vuilleumier P. (2010). Supramodal representations of perceived emotions in the human brain. Journal of Neuroscience.

[bib44] Redcay E., Courchesne E. (2008). Deviant functional magnetic resonance imaging patterns of brain activity to speech in 2–3-year-old children with autism spectrum disorder. Biological Psychiatry.

[bib45] Richter W., Richter M. (2003). The shape of the fMRI BOLD response in children and adults changes systematically with age. NeuroImage.

[bib46] Rutter M., Bailey A., Lord C. (2003). Social communication questionnaire (SCQ).

[bib47] Sanchez C.E., Richards J.E., Almli C.R. (2012). Neurodevelopmental MRI brain templates for children from 2 weeks to 4 years of age. Developmental Psychobiology.

[bib48] Schultz R.T. (2005). Developmental deficits in social perception in autism: the role of the amygdala and fusiform face area. International Journal of Developmental Neuroscience.

[bib49] Stewart M.E., McAdam C., Ota M., Peppe S., Cleland J. (2013). Emotional recognition in autism spectrum conditions from voices and faces. Autism.

[bib50] Taylor S.E., Eisenberger N.I., Saxbe D., Lehman B.J., Lieberman M.D. (2006). Neural responses to emotional stimuli are associated with childhood family stress. Biological Psychiatry.

[bib51] Warren J.E., Sauter D.A., Eisner F., Wiland J., Dresner M.A., Wise R.J.S. (2006). Positive emotions preferentially engage an auditory-motor “Mirror” system. Journal of Neuroscience.

[bib52] Zwaigenbaum L., Bryson S., Rogers T., Roberts W., Brian J., Szatmari P. (2005). Behavioral manifestations of autism in the first year of life. International Journal of Developmental Neuroscience.

