# Supplementary material for: Atypical processing of voice sounds in infants at risk for autism spectrum disorder
Source: Cortex. 2015 Oct;71:122–33. doi: 10.1016/j.cortex.2015.06.015 (PMC4582069; doi:10.1016/j.cortex.2015.06.015)
Supplement: Supplementary file 1 [file mmc1.docx]

**Table 1**. Description of the main dimensions of maternal and infant behaviours coded using the Global Rating Scales (Murray *et al*., 1996)

| **Dimension*** | **Item clustering on the GRS** | **Definition** |
| --- | --- | --- |
| **Mother** |  |  |
| Sensitivity | *(i) Warm-Positive, (ii) Accepting-Rejecting, (iii) Responsive-Unresponsive, (iv)Demanding-Non-demanding, (v) Sensitive-Insensitive* | Measurement of the quality of maternal interaction on a scale of sensitive to insensitive |
| Intrusiveness | *(i)Non-intrusive-Intrusive, (ii) Non-intrusive-Intrusive speech* | Interruption of the infant’s activity either verbally or through physical activity around the infant |
| Remoteness | *(i) Non-remote-Remote, (ii) Non-silent-Silent* | Psychological and verbal disengagement |
| Depressive affect | *(i)Happy-Sad, (ii) Much energy –Low energy, (iii) Absorbed in the infant-Self-Absorbed* | Display of depressive affect, through sadness, slowness and focus on the self rather than the infant |
| **Infant** |  |  |
| Attentiveness | *(i)Attentive-Avoidant, (ii) Active Communication-No communication, (iii) Positive vocalisations- No positive vocalisations* | Measurement of the quality of infant engagement |
| Active-Engagement | *(i)Engaged with the environment-Self-Absorbed, (ii) Lively-Inert, (iii) Attentive- Avoidant* | Level of liveliness, attentiveness to mother, and interest in the environment and /or caregiver. |
| Fretfulness | *(i)Happy-Distressed, (ii) Non-fretful-Fretful* | Measurement of infant distress and irritability |

Note: * Composite dimension scores comprise an average of the scale items. Items are scores on a rating scale of 1-5; higher scores indicate more positive behaviours (for e.g. increased maternal sensitivity

**Table 2**. Clusters with significant voice-sensitivity (neutral voice > non voice) within each group.

|  | BA | |  | Tal(x) | | Tal(y) | Tal(z) | Effect size | N voxels | Mass |
| --- | --- | --- | --- | --- | --- | --- | --- | --- | --- | --- |
| LR Group | |  | | |  | | |  |  |  |
| L Fusiform Gyrus | 20 | |  | -50.56 | | -29.63 | -29.15 | 0.005779 | 3 | 8.01 |
| R Superior Temporal Gyrus | 38 | |  | 50.56 | | 3.7 | -12.65 | 0.002719 | 4 | 12.11 |
| L Middle Temporal Gyrus | 21 | |  | -61.39 | | 0 | -7.15 | 0.000818 | 13 | 42.45 |
| R Superior Temporal Gyrus | 22 | |  | 57.78 | | -22.22 | 3.85 | 0.005308 | 5 | 13.1 |
| L Middle Frontal Gyrus | 8 | |  | -46.94 | | 14.81 | 42.35 | 0.008495 | 3 | 7.55 |
| L Superior Frontal Gyrus | 8 | |  | -21.67 | | 29.63 | 47.85 | 0.012007 | 6 | 14.77 |
| R Cingulate Gyrus | 24 | |  | 14.44 | | -3.7 | 47.85 | 0.000315 | 19 | 56.55 |
| HR Group | |  | | |  | | |  |  |  |
| R Inferior Parietal Lobule | 40 | |  | 54.17 | | -29.63 | 36.85 | 0.000094 | 35 | 102.23 |
| R Cingulate Gyrus | 24 | |  | 7.22 | | -14.81 | 42.35 | 0.000713 | 6 | 19.7 |

**Table 3**. Clusters with significant non voice > neutral voice contrast within each group.

|  | BA | |  | Tal(x) | | Tal(y) | Tal(z) | Effect size | N voxels | Mass |
| --- | --- | --- | --- | --- | --- | --- | --- | --- | --- | --- |
| LR Group | |  | | |  | | |  |  |  |
| R Uncus | 34 | |  | 10.83 | | 0 | -23.65 | 0.002951 | 4 | 11.99 |
| L Inferior Frontal Gyrus | 47 | |  | -21.67 | | 7.41 | -18.15 | 0.00541 | 7 | 18.17 |
| L Middle Temporal Gyrus | 37 | |  | -43.33 | | -59.26 | 3.85 | 0.004601 | 4 | 9.79 |
| R Thalamus |  | |  | 21.67 | | -18.52 | 3.85 | 0.007104 | 6 | 16.42 |
| R Putamen |  | |  | 21.67 | | 3.7 | 14.85 | 0.001494 | 17 | 49.53 |
| HR Group | |  | | |  | | |  |  |  |
| L Cerebellum |  | |  | -25.28 | | -33.33 | -34.65 | 0.005526 | 8 | 18.87 |
| L Inferior Temporal Gyrus | 20 | |  | -46.94 | | -22.22 | -34.65 | 0.00596 | 7 | 18.78 |
| R Precentral Gyrus | 6 | |  | 32.5 | | 7.41 | 31.35 | 0.009959 | 6 | 15.68 |


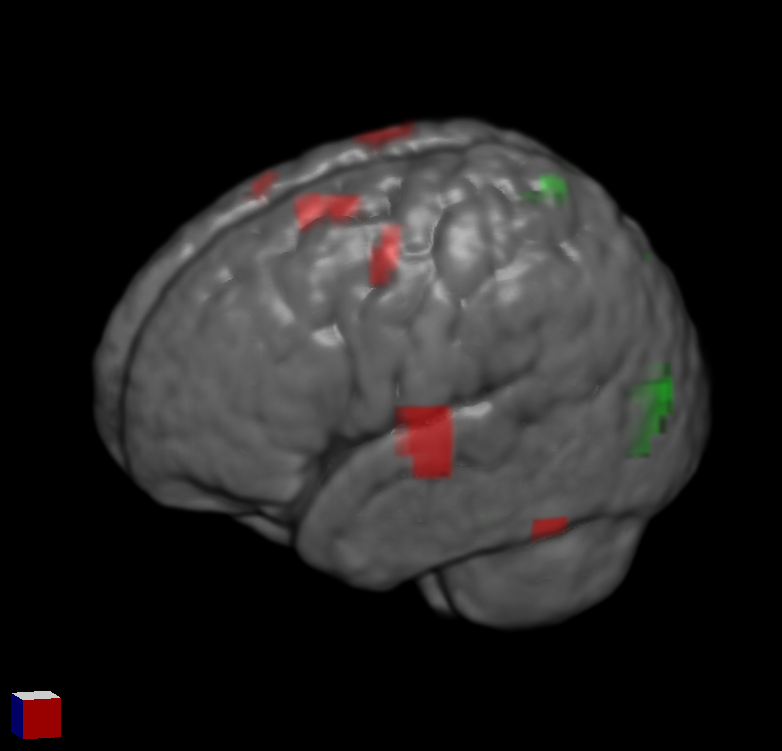

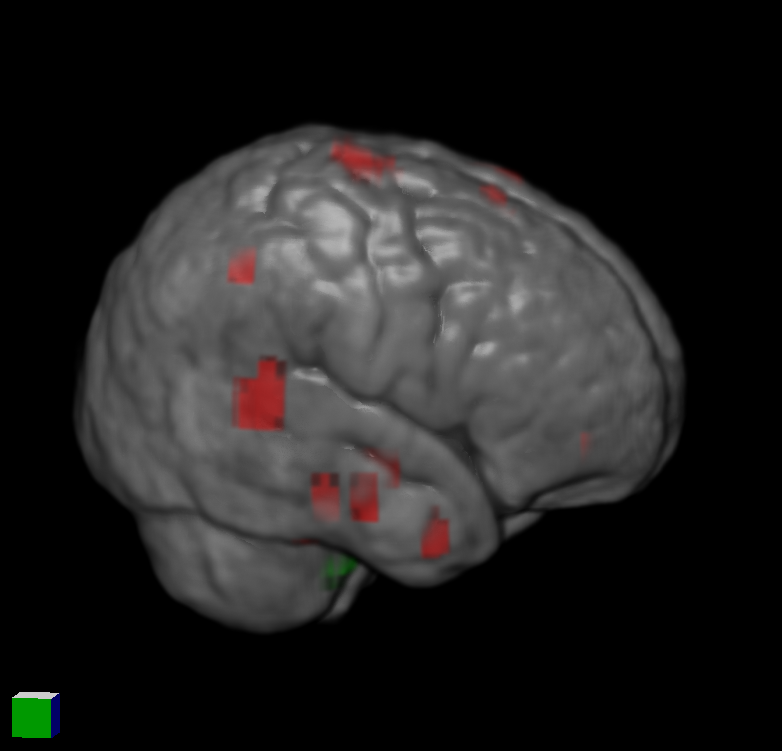


(a)


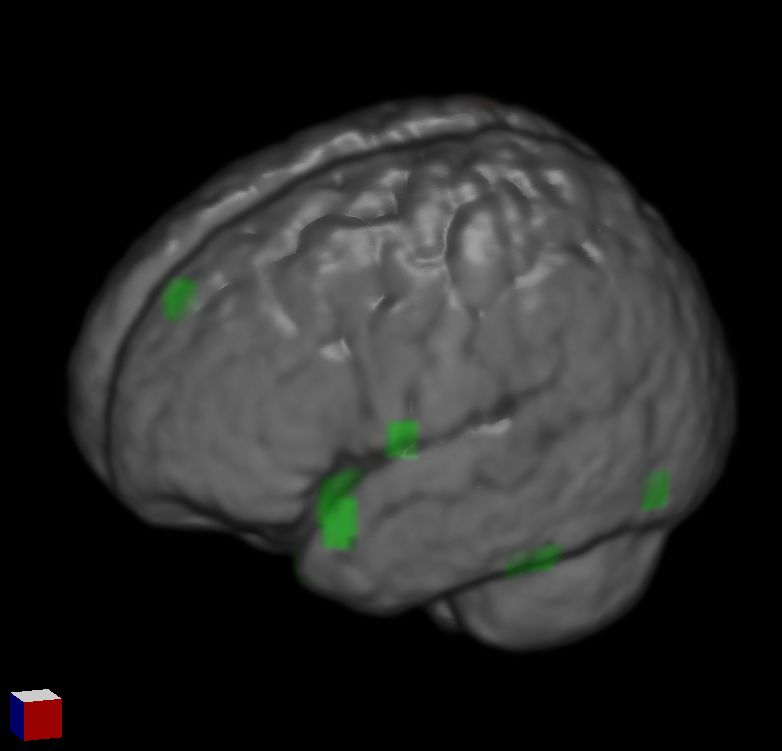

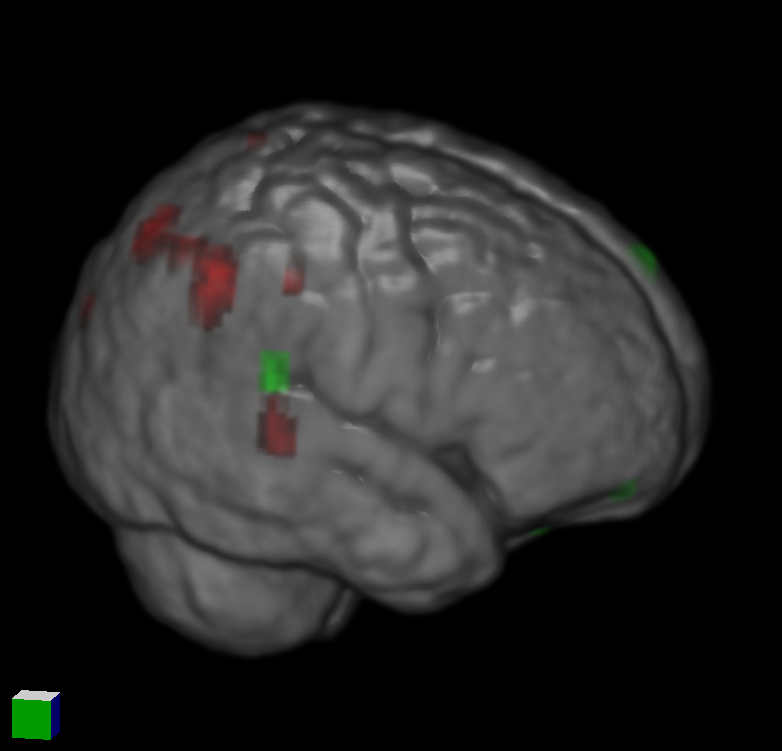


(b)

**L**

**R**

**R**

**L**

**Figure 1.** Representation on an age-appropriate infant template (Sanchez *et al*., 2012) of the neutral voice versus non voice condition contrasts: Neutral Voice > Non voice (red) and Non Voice > Neutral Voice (green). (a) low-risk group, (b) high-risk group. Left (L) and right (R) hemispheres. See also Supplementary Tables 1 and 2, and Figure 1.

**Table 4**. Clusters with significant sensitivity to sad affect in voice (sad voice > neutral voice) within each group.

|  | BA | |  | Tal(x) | | Tal(y) | Tal(z) | Effect size | N voxels | Mass |
| --- | --- | --- | --- | --- | --- | --- | --- | --- | --- | --- |
| LR Group | |  | | |  | | |  |  |  |
| L Superior Frontal Gyrus | 10 | |  | -29 | | 70 | -7 | 0.012602 | 3 | 7.2 |
| R Inferior Frontal Gyrus | 45 | |  | 47 | | 30 | -2 | 0.00147 | 5 | 15.5 |
| R Inferior Frontal Gyrus | 45 | |  | 58 | | 22 | 9 | 0.003669 | 4 | 11.63 |
| HR Group | |  | | |  | | |  |  |  |
| R Cingulate Gyrus | 24 | |  | 22 | | 4 | 37 | 0.0024 | 8 | 22.92 |

**Table 5**. Clusters with significantly stronger responses to neutral voice compared to sad voice, within each group.

|  | BA | |  | Tal(x) | | Tal(y) | Tal(z) | | Effect size | | N voxels | | Mass | |
| --- | --- | --- | --- | --- | --- | --- | --- | --- | --- | --- | --- | --- | --- | --- |
| LR Group | |  | | |  | | |  | |  | |  | |  |
| R Uncus | 38 | |  | 29 | | 4 | -40 | | 0.001489 | | 7 | | 20.68 | |
| R Superior Temporal Gyrus | 21 | |  | 58 | | -22 | -2 | | 0.011697 | | 3 | | 7.79 | |
| L Middle Frontal Gyrus | 9 | |  | -43 | | 19 | 31 | | 0.00449 | | 6 | | 16.6 | |
| L Middle Frontal Gyrus | 8 | |  | -22 | | 26 | 37 | | 0.003305 | | 9 | | 22.16 | |
| HR Group | |  | | |  | | |  | |  | |  | |  |
| L Fusiform Gyrus | 37 | |  | -36 | | -41 | -13 | | 0.000517 | | 7 | | 23.08 | |
| R Fusiform Gyrus | 20 | |  | 43 | | -37 | -18 | | 0.002271 | | 9 | | 23.25 | |
| R Fusiform Gyrus | 37 | |  | 40 | | -59 | -7 | | 0.000168 | | 3 | | 10.6 | |
| R Fusiform Gyrus | 19 | |  | 29 | | -59 | -7 | | 0.022397 | | 4 | | 8.37 | |
| R Lingual Gyrus | 18 | |  | 11 | | -74 | 4 | | 0.007658 | | 5 | | 12.7 | |
| R Middle Frontal Gyrus | 10 | |  | 33 | | 63 | 9 | | 0.001075 | | 5 | | 14.59 | |
| L Precentral Gyrus | 6 | |  | -47 | | -11 | 26 | | 0.0328 | | 5 | | 10.13 | |

**Fig 2.** Beta values averaged across participants from each group from the three contrasts of interest. Individual beta values per contrast were extracted from the clusters where significant group differences were detected in the corresponding contrast of interest, then averaged across participants within each group. Contrasts: (a) Voice > Non Voice, (b) Sad > Neutral Voice and (c) Neutral > Sad Voice. In the y-axis, the averaged beta values; in the x-axis, the clusters of interest identified by ID (in parentheses) and by brain region, where L or R indicate left or right hemisphere. Abbreviations from brain regions are included in the chart.

**Supplemental Experimental Procedures**

**Stimuli**

Voice stimuli were chosen from the Montreal Affective Voices [28], the stimuli of the functional localizer of the ‘Temporal Voice Areas’ available on the Voice Neurocognition Laboratory website (http://vnl.psy.gla.ac.uk/resources_main.php). Some Non Voice stimuli were chosen from the Voice Neurocognition Laboratory website (water sounds), while others were recorded by the authors (toy sounds). Each stimulus sequence lasted 21 seconds and consisted of 7-11 different sounds interleaved by short periods of rest (between 0.47 and 0.75 seconds). Voices were produced by different adult speakers (male and female). In each 21 seconds stimulus sequence, the sound volume was gradually increased over a period of 6 seconds to avoid a startle response. A block design was used to maximize statistical power, in which 21 seconds of auditory stimuli were alternated with 9 seconds of rest. A complete fMRI session comprised 32 blocks (8 in each stimulus category) for a total of 16 minutes. The order of stimulus presentation was weighted so that the neutral vocalization and Non Voice categories appeared more frequently at the beginning of each session. The presentation order aimed to maximize the amount of data in the Voice versus Nonvoice contrast in case an infant could not complete the whole fMRI session.

**Testing Procedure**

The scanning sessions were organised around the infant’s nap time and families were invited to come to the Imaging Centre with plenty of time to settle in. When asleep, the infant was swaddled in a cotton sheet, and comfortably positioned in a MedVac Vacuum Immobilization Bag (DFI Medical Solutions), to reduce movement during scanning. Natus MiniMuffs Noise Attenuators were placed on the infant’s ears and the scanner bore was lined with sound attenuating foam in order to reduce the scanner noise perceived by the infant. In addition, MR-compatible piezoelectric headphones (http://www.mr-confon.de/en/) were placed on top of the MiniMuffs to reduce residual noise from the MRI scanner and present the stimuli. The sound level of the stimuli was adjusted to a comfortable level for the infants, but that was loud enough to be heard above the residual scanner noise and the MiniMuffs sound attenuation.

MRI data were acquired using a GE 1.5 Tesla Twinspeed MRI scanner (General Electric, Milwaukee, WI, USA). 320 T2* weighted gradient echo planar multi-slice datasets depicting BOLD (Blood Oxygenation Level Dependent) contrast were acquired in each of 24 non-contiguous near-axial planes (4.0 mm thick with 1.0 mm spacing, 3.5 x 3.5mm in-plane resolution) parallel to the Anterior Commissure-Posterior Commissure (AC-PC) line (TE 57 ms, TR 3000 ms, flip angle 90°, number of signal averages = 1, 16:04 minutes). At the same session a T2 weighted fast spin echo (FSE) dataset was acquired (256x168 rectangular matrix, 2mm slice thickness, 0mm slice gap, field of view=18cm, TR=4500, TE=113ms, echo train length=17). Gradient rise times were limited in order to reduce the noise of the pulse sequences to approximately 70dB. Daily quality assurance was carried out to ensure high signal to ghost ratio, high signal to noise ratio and excellent temporal stability using an automated quality control procedure (Simons et al., 1999). The body coil was used for RF transmission and an 8-channel head coil for RF reception procedure (Simons et al., 1999).

**Data Analysis**

The data was analysed with XBAM software (www.brainmap.co.uk/xbam.htm) using a data-driven approach. Minimization of motion-related artefacts and removal of linear trends was carried out with a rigid-body transform to account for translation and rotation using a spin history correction (Bullmore et al., 1999). After registration, the 3D images were realigned at each time point by finding the combination of rotations (around the three axes) and translations (in three dimensions) that maximized the correlation with an image obtained by averaging the intensity at each voxel over the whole experiment. Then, data were smoothed using a Gaussian filter (7.2 mm isotropic FWHM). Each component of the experimental design was convolved with two gamma variate functions (peak responses at 4 and 8 sec respectively). Then, the best fit between the weighted sum of these convolutions and the time series at each voxel was computed by standard general linear modelling (GLM) and an estimate of the response (beta) obtained for each experimental condition. As reported in our previous publication (Blasi et al., 2011), instead of the standard adult HRF, we adopted a strategy of obtaining HRF information directly from the data, whilst minimizing the statistical bias that could result from this approach. Reasoning that an auditory experiment should produce a dominant auditory response, we obtained the HRF in the auditory cortex for each subject by deconvolution from the mean time-series response in this brain region. For each subject, we then used the mean HRF estimated from all the other subjects, thus producing the best estimate of the HRF unbiased by the subject being analysed. The data for each subject were then analysed using standard GLM analysis and the estimated unbiased HRF. This was repeated for all participants. In the last step of the data analysis, the data were normalized to Talairach space using an infant template as previously described by (Dehaene-Lambertz et al. 2002), and similarly to our previous publication (Blasi et al. 2011). As an infant template was used, the Talairach coordinates provided in the Tables must be treated with caution as they are only an approximation to the adult coordinates.

After normalization, a one sample t statistic was computed at each voxel using the beta estimates for each individual. The significance of this t statistic was then tested at a voxel-wise level by data permutation. Briefly, the signs of the betas were randomly permuted and the resulting permuted betas used to recalculate a one-sample t statistic. Repeating this procedure 40,000 times per voxel produces the distribution of t under the null hypothesis without the requirement for the data to follow a normal distribution – an assumption often violated in small group fMRI data. The significance of the t obtained from the unpermuted data was assessed by reference to the probability distribution of t obtained by data permutation. We used an uncorrected p value of 0.005 for voxel-wise maps and we report activation in clusters as small as 3 voxels.

**Supplemental references:**

Simmons, A., Moore, E., and Williams, S.C. (1999). Quality control for functional MRI using automated data analysis and Shewhart charting. Magnetic Resonance in Medicine, 41, 1274-1278.

Bullmore, E.T., Brammer, M.J., Rabe-Hesketh, S., Curtis, V., Morris, R.G., Williams, S.C.R., Sharma, T., and McGuire, P.K. (1999) Methods for diagnosis and treatment of stimulus correlated motion in generic brain activation studies using fMRI. Human Brain Mapping 7,38-48.
